# Clinical Lectures on Diphtheria

**Published:** 1864-06

**Authors:** John T. Metcalfe

**Affiliations:** Prof. Practice of Medicine, University Medical College


### CLINICAL LECTURES ON DIPHTHERIA.

By JOHN T. METCALFE, M. D.,
*Prof. Practice of Medicine, University Medical College.*

LECTURE I.

I will occupy your time this morning, gentlemen, by some remarks on two cases that have lately presented themselves to us, at our Wednesday's clinic.

The first has the following history, which I now read to you from the case-book :—

Margaret Johnson, seven years old, a well developed child for her age, came before the class complaining of loss of power over the vocal organs, and difficulty of deglutition.

Her mother stated that she had been in good health until three months before coming to the college, when she observed Margaret to be troubled in swallowing her food. Not only was the act performed with dtfficuly and with occasional apparent pain, but the fluid portions of her food would return through the nostrils, when the pharyngeal muscles contracted. Now and then, in vomiting, the food would pass as much through the nose as through the mouth. The child's intellect was apparently in a normal condition. She readily understood everything that was said to her, but lacked the power of intelligible expression.

Soon after the symptoms above noted made their appearance, the patient complained of torpid bowels, of dysuria, and loss of power over the lower extremities. A few days later the arms and hands were likewise affected, and it was observ- that the sight became less distinct than usual. There was convergent strabismus of the left eye. Hearing unaffected. Lately she she has slept uneasily, and snores heavily and continuously when asleep. At present the partial paralysis above noted exists, but without marked increase. The skin is cool; the pulse quicker than natural; the head drops forward on the chest, and can not be raised by voluntary effort. On each side of the neck the lymphatic glands, especially about the angle of the jaw, are enlarged. Conformation of mouth and tongue normal; pharynx red, but without membrane. On questioning the mother closely she told us, you remember, that Margaret had been ailing for some days before the symptoms above mentioned had manifested themselves. She had had pain in deglutition, as evinced by the necessity of drawing the head downwards and forwards, and making a sort of grimace as one with a sore throat would do. She had been fretful, restless, weak, and without her usual appetite.

Another case which came before us a few days ago, I find thus noticed in Mr. James Moore's notes:—

Timothy Quinlan, æt. 8 years, came to the Clinic for difficulty about the vocal apparatus.

He has never been a robust child, but has never had any serious illness previously. His parents are people of good constitution, without hereditary or other disease. About a month ago, his mother observed him to be troubled with sore throat, but he had no apparent fever nor loss of appetite. Under the angle of the jaws she noticed sensitive lumps, as large as a hickory nut, which lasted until a physician was called in, who gave him medicine. This, his mother thinks, cured the lump and sore throat. His deglutition was painful.

During the time referred to, lasting about a week, the boy was troubled by a harsh, dry, cough, with great hoarseness and indistinctness of the voice, so that it was difficult to understand him in ordinary conversation; now he speaks like a person with cleft palate, and, according to his mother, his intelligibility is diminishing from day to day. His throat, on examination, shows great redness of the fauces, with apparent thickening of the mucous membrane, especially of the right side. There is a pendulous condition of the soft palate, as though the muscles supporting it had lost their tone. There is double converging strabismus. His pulse is natural; skin warm and slightly moist. Anxious expression of countenance. On attempting to cough, he is unable to make the short, sharp, explosive expiration which should constitute the act. It is more like a harsh, *blowing* sound. His appetite is not bad; bowels regular; urine not examined.

These cases, gentlemen, are interesting, as illustrating some points in the pathology of a disease which has given great anxiety to the medical profession in this country as well as in Great Britain, during the last few years.

Diphtheria is not a new disease, as many have supposed—partly from the new name by which it is now known, and for which it was indebted to Mr. Farre, the Registrar-General of England, and partly because many physicians of large experience have only of late met with it as an epidemic malady,

To the excellent monograph of Dr. Edward Heanlam Greenhow, and to the admirable lectures of Prof. Alonzo Clark, published in last year's *Medical Times and Gazette*, I refer you for much interesting matter concerning its history.

Most of us who have practiced fifteen or twenty years, will easily remember sporadic cases of diphtheria. It is only as an epidemic that it has been comparatively unknown.

I shall, in the first place, speak to you of the peculiar sequelæ, so well illustrated in our two patients—I mean the diphtherial paralysis—and will then endeavor to anticipate answers to such letters as I am constantly receiving from our graduates who have commenced practice, and, having fallen in with the disease under consideration, desire to know what I consider the best general treatment.

"Nerve affections following diphtheria consist chiefly of impaired, perverted, or excessive sensibility, together with a more or less complete paralysis of the muscles of the fauces, pharynx, tongue, lips, extremities, trunk, and neck. The frequency of the occurrence of these symptoms in the several sets of muscles being nearly in accordance with the order in which they have been named, those of the fauces being the most frequent, and those of the neck least so." Doctor Greenhow,from whose article in the August (1863) number of the *Edinburgh Medical and Surgical Journal* I make the foregoing extract, does not mention paralysis of the eye muscles. This is by no means unfrequent. It existed in the two cases I have mentioned to you, but is not relatively so common as they might lead you to suppose. Perhaps in order of frequency we might place it after that of the trunk.

The supervention of diphtherial paralysis varies much with regard to the time of its access. In the cases which I have exhibited to you, there has appeared, in one, to be scarcely any appreciable interval between the primary and secondary affections. But this is not the general rule. As we ordinarily see it, the loss of muscular power has come on after apparent convalescence from diphtheria proper. In some instances, patients have been removed from the physician's immediate care to convalesce in some distant place, after which the paralytic symptoms have developed themselves—in one case as long as two months after the first attack.

The practical deduction to be drawn from these facts is to avoid pronouncing your patient cured until several weeks have elapsed after apparent recovery. It is a general rule that the probability and extent of paralysis bear a direct ratio to the intensity with which the antecedent diphtheria has manifested itself; and this latter may serve also to measure the extent to which loss of muscular power will go.

Nevertheless, this is a rule subject to numerous exceptions, as the cases referred to in this lecture will show. Indeed, in one of them it would not have occurred to a physician's mind, before our knowledge of diphtheria was as accurate as it has become of late years, to make any connexion between the slight attack of sore-throat and the faucial and other paralysis. In the boy's case, the child was not even obliged to go to bed, and continued to enjoy a good appetite during his apparently trivial indisposition. In some of the worst cases of diphtheria I have ever seen, there was no subsequent paralysis of any sort.

Among the sequelæ most calculated to cause anxiety on the patient's part is an apparent loss of sight. This depends, not on any organic affection of the retina, but is a muscular affair—the ciliary muscle being the one involved in paralysis. Patients will occasionally be unable to read the print of an ordinary book, who will yet clearly distinguish objects across a hospital ward. The use of appropriate glasses will often restore the power of vision.

The history of our patients will show you that the paralytic troubles are generally progressive; that is, they may attack any one part of the muscular system, increase in intensity, and subsequently travel, without any seeming rule, to others. Usually gradual in its development, the paralysis may exceptionally be sudden.

It is not always regular in its march. Thus, in one of my late cases, I would find the child able to use her arms and legs fairly on one day, on the next there would be much less ability to do so. On the following visit she would have undergone great improvement. So it was with the speech and with deglutition. Later in the disease convalescence became steady and uninterrupted.

In the patients whom you have seen there was no mention made of anæsthesia. This is not an unfrequent phenomenon. We may have it manifested in any part of the body. Usually I have observed it most markedly in the faucial and palatine mucous membrane. It follows the course of the muscular paralysis, and requires no especial consideration.

The same may be said of hyperæsthesia. In the course of convalescence from the paralytic sequelæ, there will now and then be much complaint from excessive tenderness of the affected muscles on pressure being made.

In some cases the patient complains of the skin being so tight as to give the sensation of a swollen member. This is merely a perversion of sensibility, as inspection will show no change in size, nor to the observer's touch will there be anything abnormal.

Among all varieties of diphtherial paralysis, the most interesting and the most dangerous is that in which the heart is the organ concerned. At times the pulse will grow more and more feeble and infrequent, until it finally ceases to be perceptible. In the case of a young lady to whom I was called, in New Jersey, death was due to almost instantaneous cessation of the heart's action. She was apparently in satisfactory convalescence, when on walking across the sitting-room, she suddenly dropped down and died. There was no previous organic disease of the heart, nor was there any brain lesion that would explain her abrupt death.—*Am. Med. Times.*

